# Biomechanical Assessment of Syndesmotic and Deltoid Ligament Strain in Pronation-External Rotation Type Ankle Injuries by Musculoskeletal Computer Simulation

**DOI:** 10.1177/24730114261423185

**Published:** 2026-03-06

**Authors:** Ola Saatvedt, Mohammad Amin Shayestehpour, Øystein Bjelland, Martin G. Gregersen, Håvard Furunes, Marius Molund

**Affiliations:** 1 Division of Orthopaedic Surgery, Oslo University Hospital, Norway; 2 University of Oslo Faculty of Medicine, Oslo, Norway; 3 Department of Orthopaedic Surgery, Østfold Hospital Trust, Grålum, Norway; 4 Department of ICT and Natural Sciences, Norwegian University of Science and Technology, Ålesund, Norway; 5 Department of Research and Innovation, Møre and Romsdal Hospital Trust, Ålesund, Norway; 6 Department of Physical Medicine and Rehabilitation, Østfold Hospital Trust, Grålum, Norway; 7 Department of Orthopedic Surgery, Innlandet Hospital Trust Gjøvik Hospital, Gjovik, Norway

**Keywords:** ankle fracture, Lauge-Hansen, biomechanics, deltoid ligament

## Abstract

**Background:**

Suprasyndesmotic ankle fractures, commonly resulting from pronation-external rotation (PER) mechanisms, are traditionally associated with disruption of the syndesmotic ligaments, a medial malleolus fracture or complete deltoid ligament rupture. However, recent imaging and clinical studies suggest that key stabilizing ligaments may remain intact in certain cases, potentially affecting talocrural stability. This pilot study aims to evaluate modelled ligament tension patterns in PER injuries using a validated musculoskeletal computer simulation model.

**Methods:**

A musculoskeletal model of the ankle joint was developed using the AnyBody Modeling System (version 7.4), incorporating detailed anatomical structures and ligament biomechanics. The PER mechanism was simulated by applying external rotation (0-50 degrees) with the foot fixed, and ligament tensional forces was recorded for the deltoid and syndesmotic complexes. Because of limitations in the computer model, a fibular fracture was not simulated. Strain patterns were analysed across simulation steps to assess the sequence and magnitude of ligament loading.

**Results:**

The anterior and superficial deltoid ligaments (tibionavicular, deep anterior tibiotalar, tibiospring, and tibiocalcaneal) and the anterior inferior tibiofibular ligament (AITFL) demonstrated early and substantial increases in tension. Conversely, the deep posterior tibiotalar ligament (dPTTL) and posterior inferior tibiofibular ligament (PITFL) showed minimal strain during the majority of the simulation, suggesting they may remain intact in a subset of PER injuries.

**Conclusion:**

In a computer-simulated pronation-external rotation injury mechanism, the observed tensional force acting on the posterior inferior tibiofibular ligament and the deep posterior tibiotalar ligament is substantially lower compared to the remaining ligaments of the syndesmotic and deltoid complex. The study highlights the potential of a of a novel computer-based ankle/foot model as an alternative to traditional in vitro biomechanical studies.

**Clinical Relevance:**

This study finds that in a simulated pronation-external rotation injury to the ankle, key stabilizing ligaments show low tensional forces, suggesting they might be spared from complete rupture. These findings challenge traditional views of complete ligament disruption in PER injuries, and may question the injury cascade originally described by Lauge-Hansen. Additionally, it highlights the use of computer simulation as an alternative to traditional biomechanical research and offers new opportunities for hypothesis generation, improving diagnosis, and injury classification.

## Introduction

Suprasyndesmotic ankle fractures typically occur in a pronation-external rotation type (PER) injury mechanism as first described by Lauge-Hansen.^1^ These fracture types account for approximately 10% of all ankle fractures, and the incidence is increasing.^2,3^ The injury cascade described by Lauge-Hansen^1^ in their original experiment results in a complete disruption of the medial deltoid and syndesmotic ligament integrity (Table 1). Because of the extent of ligamentous injuries associated with these fractures, operative treatment is indicated to reestablish stability of the ankle joint.^4^ However, radiographs and clinical tests often fail to identify significant ligamentous injuries. These challenges may be addressed with a deeper understanding of the sequency and extent of ligamentous injury associated with these fractures.

**Table 1.** Lauge-Hansen Pronation Injuries.

Stage	PER
I	Deltoid rupture or medial malleolus fracture
II	AITFL and IOL rupture
III	Suprasyndesmotic fibula fracture
IV	PITFL rupture or Posterior malleolus fracture

Abbreviations: AITFL, anterior inferior tibiofibular ligament; IOL, interosseous ligament; PER, pronation-external rotation; PITFL, posterior inferior tibiofibular ligament.

Recent clinical studies on transsyndesmotic/Lauge-Hansen supination-external rotation (SER) fractures highlight the importance of the deltoid ligament for talocrural stability and its role in non-operative treatment of ankle fractures.^5-8^ Furthermore, ongoing research suggests that a subset of suprasyndesmotic/PER fractures may have intact medial attachments, potentially allowing for non-operative treatment.^9^ In a recent publication on magnetic resonance imaging (MRI) findings in weightbearing stable suprasyndesmotic ankle fractures, the deep posterior tibiotalar ligament, and the posterior inferior tibiofibular ligament were found to be rarely completely ruptured.^10^ These findings challenge the traditional injury cascade associated with these fractures and may affect the stability of the talocrural joint. Based on our findings in the recent MRI study on similar injuries, we hypothesize that in a computer-simulated PER mechanism the tension affecting the dPTTL and the PITFL is low compared with the remaining stabilizing ligaments of the ankle joint.

Biomechanical cadaveric studies are time and resource consuming, and in some cases the access to appropriate cadavers is limited. With the evolution of computer science, new methods of doing biomechanical analysis and testing are evolving.^11-13^ AnyBody Modeling System (version 7.4) is a musculoskeletal modelling and simulation tool that enables biomechanical testing with precise and dynamic prediction of ligament tension.^14^ A recent validation study on Weber B/SER injuries demonstrates strong correlation when compared to a cadaveric model and forms the basis for a novel method of biomechanical testing of the foot and ankle.^15^

By employing the AnyBody simulation tool, our aim with this pilot study is to examine the tension of the syndesmotic and deltoid ligament complex in a pronation external-rotation type injury mechanism. A deeper understanding of the ligamentous reactions associated with different injury mechanisms may aid in predicting injury patterns and their effect on stability.

## Method

**Figure 1.**
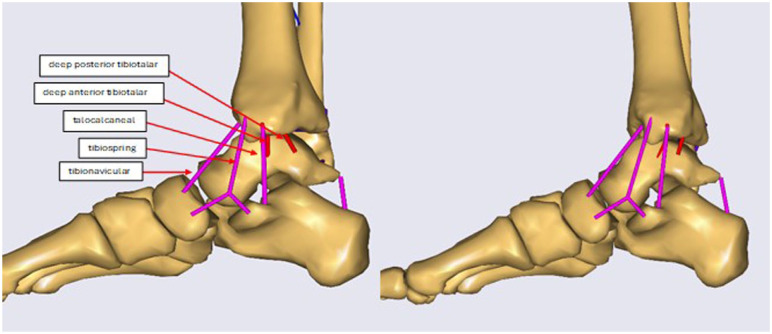
Sagittal view of the deltoid complex at the neutral position (left) and the last step, fully rotated (right).

**Figure 2.**
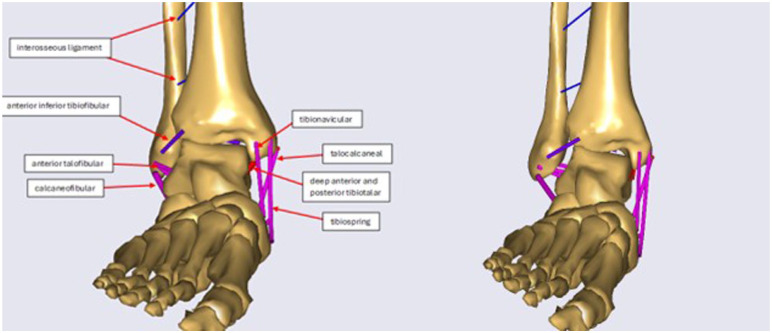
Anteroposterior view of syndesmotic and deltoid ligament in neutral position on the left and the last step (fully rotated) on the right.

The musculoskeletal model of the ankle joint was developed using the AnyBody Modelling System (version 7.4).^14^ Bones, joints, tendons, and ligaments are available in the model (Figures 1 and 2). Ligamentous anatomy and biomechanics have been derived from previous anatomic, biomechanical, and experimental studies and incorporated in the model. Based on the validation study, a passive model was selected.^15^ A passive model is equivalent to a cadaveric sample where the muscles are not active, and therefore, only the passive structures, especially the ligaments, stabilize the model.

The ankle joint model includes the talus, tibia, and fibula as separate bones, and the remaining bones of the hindfoot as a single rigid unit. The model has 3 anatomical joints—tibiofibular, subtalar and talocrural—and a simulated joint between the environment and the foot to ensure proper setup (Figure 1 and 2). Unlike traditional models that simplify the ankle joint to 1 degree of freedom, this model includes several degrees of freedom (DOF) for the talocrural, subtalar, and tibiofibular joint, allowing individual movements in various directions for the individual joints. To simulate the experiments as accurately as possible in the model environment, the robot’s dynamics were simulated as a force and a moment on top of the tibia, and the foot fixed to the ground. The model was developed and validated using experimental data based on SER injury experimental data. For a detailed explanation of the model see supplemental materials (Table S1-Table S4).

The ligaments in the model include the lateral ligaments—anterior talofibular (ATFL), calcaneofibular (CFL), and posterior talofibular (PTFL)—and the medial ligaments—deep posterior tibiotalar (dPTTL), tibiocalcaneal (TCL), tibiospring (TSL), deep anterior tibiotalar (dATTL), and tibionavicular (TNL). The posterior talocalcaneal (PTCL) is an additional ligament not a part of either group. The tibiofibular syndesmotic ligaments, including the interosseous ligament (IOL), anterior tibiofibular ligament (ATIFL), and posterior tibiofibular ligament (PTIFL), were also defined (Figures 1 and 2).

Ligament tensional forces across the talocrural joint with different movements was measured and compared to the sequence as described by Lauge-Hansen in their original article (see Table 1). Ligament output was recorded as tensile force (N), consistent with prior cadaveric and computational studies, allowing comparison with published ligament load-to-failure data.^16^ In the present model, joint reaction forces and ligament tensile forces are solved concurrently and represent complementary components of the overall mechanical response rather than competing forces. The distal tibiofibular and talocrural joints allows controlled relative motion, whereas the ligaments are modelled as passive elastic structures that develop tensile force in response to elongation. During externally applied rotation, ligament forces arise from relative intersegmental motion, and joint reaction forces represent the net forces required to maintain joint stability and equilibrium. To simulate a PER mechanism without complicating the model, the external rotation of the joint was the only degree of freedom that was controlled manually, and the rest of the movements were consequential rotations occurring as a result of the multi-dimensional freedom of the joints. The simulation initiates with the talocrural joint in 20 degree dorsi-flexion and zero degree pronation and external rotation. The main forced movement of the ankle joint is the external rotation from 0 to 50 degrees. However, as the nature of the ankle joint is a complex multi-dimensional movement, flexion and pronation will also change to stabilize the joint. External rotation from 0 to 50 degrees was applied to capture the complete range of ligament loading associated with external rotation ankle injuries and is in line with Lauge-Hansen’s original experiment. This range exceeds reported thresholds for syndesmotic and deltoid ligament failure, ensuring that maximal ligament strain and force responses were identified for all stabilizing structures.^1,16^

Simulation of a fibular fracture, commonly occurring in PER injuries, was not possible to re-create in the current model.

### Statistical Analysis

A graphical analysis was conducted, showing tensional force (Newton) on the *y* axis, with the *x* axis showing the rotational steps as it progresses through the simulation.

Analyses were carried out using Microsoft Excel and programmed statistics in Python (version 3.13.1).

## Results

The ligament tensions were extracted from the model per step in the simulation.

**Figure 3.**
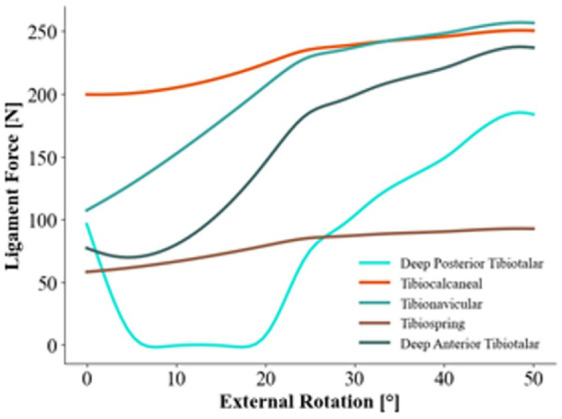
Ligament tension in newtons (N) of the individual bands of the deltoid ligament complex in a pronation external rotation injury mechanism.

For the simulation of the deltoid ligament (Figure 3), the TNL and dATTL graph is consistent with a rapid increase in tension from the initiation of rotation until they reach 230 and 175 N respectively (step 33; 33 degrees external rotation and 6 degrees pronation), and then the tension curve becomes linear and flattens out. The TSL and TCL demonstrate similar curves with a gradual increase in tension from the initiation of rotation until they reach 175 and 243 N, respectively, where the curves flatten out, just after step 30 of the rotation. The TCL graph demonstrates the highest tension at stage 0 (initiation), with approximately 200 N. The simulation of the dPTTL of the deltoid complex is consistent with a decrease in tension from the initiation of rotation. The dPTTL tension decreases to zero and does not increase until step 28, where the tension increases rapidly until approximately 180 N after a short but steep increase from step 28 to 33.

**Figure 4.**
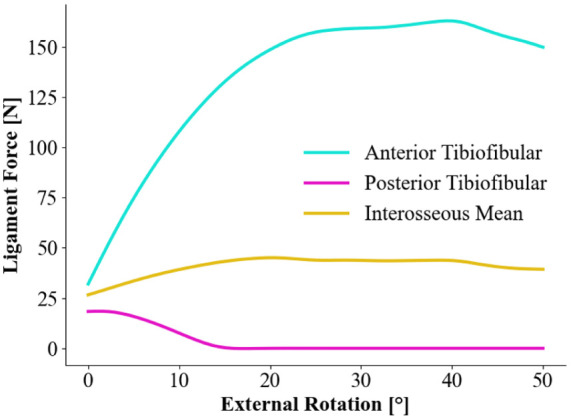
Ligament tension in newtons (N) of the syndesmotic complex during a pronation external rotation injury mechanism.

For the tibiofibular syndesmosis (Figure 4), the simulation of the AITFL demonstrates a rapid increase in tension from the initiation of rotation until it reaches a plateau from step 30 at approximately 160 N. The IOL, which is a depiction of the interosseous ligament and membrane extending from the ankle joint to the proximal one-third of the fibula, shows a gradual increase in tension from initiation and levels out with approximately 40 N of tension. In the simulation, the PITFL ligament acts inversely to the other syndesmotic ligaments with an initial decrease in tension and levels out at close to 0 N. If the rotational steps are continued to more extreme positions, the tension will eventually increase, but this is not depicted in the graph.

## Discussion

This pilot study evaluates the tensional force acting on the stabilizing ligaments of the ankle joint in a pronation-external rotation mechanism, simulated by a musculoskeletal model. The amount of tension affecting each ligament at given points throughout the rotation may give us an indication of the order of potential ligament rupture. Literature describing load-to-failure measurements until rupture of the syndesmotic and deltoid ligament complexes in PER injury mechanisms is lacking, and thus it is challenging to determine the exact tensional force where a rupture would occur. Direct comparison between simulated ligament tensile forces and experimental failure loads must be interpreted with caution because of differences in testing conditions, boundary constraints, and material assumptions. However, previously reported cadaveric failure loads for the AITFL and superficial deltoid ligament components generally range from approximately 150 to 300 N, whereas substantially higher loads have been reported for the PITFL and deep posterior deltoid ligament. In the present simulation, peak tensile forces in the AITFL and superficial deltoid ligaments approached ranges reported in experimental studies, whereas the PITFL and dPTTL remained well below commonly reported failure thresholds for the majority of the simulated rotation.^16-18^ This may imply that these ligaments are protected from rupture through the majority of the rotation and is in line with the findings from our MRI study.^10^ To our knowledge, this is the first study using a musculoskeletal computer model to biomechanically evaluate the syndesmotic and deltoid ligament tensions during a simulated rotational ankle injury.

The results suggest that the anterior/superficial parts of the deltoid ligament, AITFL and IOL, experience a steady increase in tension from the initiation of the simulated PER movement and continues throughout. These ligaments, especially the AITFL and anterior/superficial parts of the deltoid, play a crucial role in resisting external rotation of the ankle joint. These ligaments are commonly injured in external rotation mechanisms of the ankle, which the model sufficiently demonstrates, and is in line with previous biomechanical studies.^1,19^

Our findings imply that ankle pronation and external rotation exert a minimal effect on the deformation of the posterior tibiotalar ligament (dPTTL). Previous biomechanical studies have shown that external rotation protects the dPTTL by bringing the insertion points closer together and loosens the ligament.^6^ These findings contradict Lauge-Hansen’s findings, who hypothesized that the deltoid would be completely ruptured (or a medial malleolus fracture) as the first stage in PER injuries. A similar mechanism might also affect the PITFL in cases of external rotation injuries. The PITFL accounting for around 33% of the syndesmotic stability is affected to a smaller degree by the PER movement and could possibly be spared in certain physiological situations, where the deforming forces do not reach the threshold of increasing tension until a possible rupture.^20^

### Clinical Relevance and Future Applications

The surgical approach to PER injuries is rarely debated because of the expected global ligamentous disruption of the ankle joint and the risk of developing post-traumatic ankle osteoarthritis if left untreated. The evolution of radiographically stress testing ankle injuries to determine whether talocrural ligamentous stability is maintained has gained increasing interest over the last years, but the research has mainly been directed toward SER ankle injuries and ligamentous sprains.^21^ Insights from our own clinical practice and an ongoing randomized trial have prompted a reassessment of our approach to PER injuries, but evidence supporting this revaluation is currently lacking. This biomechanical pilot study supports the findings of our MRI study, suggesting that key stabilizing ligaments may remain intact in selected PER injuries. This should serve as the basis for future biomechanical and clinical studies investigating the stability of this subset of PER injuries in physiological situations.

The method of using computer simulations of injury mechanisms show promise as a novel approach to traditional biomechanical research. Computer simulations allow for the controlled manipulation of variables, providing insights into ligament behavior under various conditions that would be difficult, if not impossible, to achieve through traditional experimental methods on live tissue. As the model is further enhanced, we plan to do studies with more dynamic tests to evaluate how mimicked rupture of selected ligaments affect the stability and bony contact area in the talocrural joint. Future possibilities also include simulation of different fixation techniques on various injury patterns to evaluate stability and biomechanics.

### Model Limitations

Despite the findings of our current study, there are limitations and potential errors to the model, and therefore this should be interpreted as a pilot study conducted to serve as groundwork for future studies and generating new hypotheses.

In order to create accurate computer models, we still rely on detailed anatomical and biomechanical studies on live/cadaveric tissue, which in some cases are limited. Moreover, assumptions about ligament material properties derived from geometric characteristics may not accurately capture individual differences in the tissue. Although data on ligament properties remain scarce, the integration of data-driven methods holds potential to address these shortcomings and enhance predictive capabilities.

As the simulation only depicts modeled tensional force in each ligament, we cannot conclusively state that the order of rupture would follow the same sequence, or the potential timing of the rupture. Importantly, the model does not incorporate explicit rupture criteria or progressive ligament failure, and therefore the simulated tensile forces should be interpreted as relative indicators of ligament loading rather than predictors of exact failure timing or sequence. Although the model has been previously validated against cadaveric experimental data for rotational ankle injuries, the results should be interpreted as indicative of relative ligament loading patterns rather than absolute predictors of rupture. Accordingly, the findings should be viewed as hypothesis-generating and intended to inform future mechanical laboratory investigations. A recent biomechanical study measured the force of each individual band of the deltoid ligament with different rotational movements of the ankle, highlighting the possibility to incorporate findings from in vitro biomechanical studies into computer simulation models.^22^ In future studies, we plan to implement a dynamic model to evaluate tensional consequences of remaining ligaments after a simulated removal (rupture) of selected ligaments.

Another shortcoming is that the fractured fibula commonly seen in rotational ankle injuries is not simulated in the model, which might affect the results. The validation study did not find significant differences in measurements when comparing the simulation model (without fracture) and the cadaveric robot model (with fracture), but the effects of a potential fracture is uncertain at this time. The talonavicular joint was also not present in the model as an independent articulation, with the hindfoot represented as a single rigid segment. This simplification may influence the absolute tensile forces estimated for superficial deltoid ligament branches, including the tibionavicular and tibiospring ligaments, which span the talonavicular joint. Future iterations of the model will incorporate independent talonavicular joint motion to further refine ligament loading estimates

## Conclusion

In a computer-simulated pronation-external rotation injury mechanism, the observed tensional force acting on the posterior inferior tibiofibular ligament, and the deep posterior tibiotalar ligament is substantially lower compared with the remaining ligaments of the syndesmotic and deltoid complex. This study highlights the potential of a novel computer-based ankle/foot model as an alternative to traditional in vitro biomechanical studies, and findings should be considered hypothesis-generating and support further investigation in cadaveric and mechanical laboratory studies.

## Supplemental Material

sj-pdf-1-fao-10.1177_24730114261423185 – Supplemental material for Biomechanical Assessment of Syndesmotic and Deltoid Ligament Strain in Pronation-External Rotation Type Ankle Injuries by Musculoskeletal Computer Simulation Supplemental material, sj-pdf-1-fao-10.1177_24730114261423185 for Biomechanical Assessment of Syndesmotic and Deltoid Ligament Strain in Pronation-External Rotation Type Ankle Injuries by Musculoskeletal Computer Simulation by Ola Saatvedt, Mohammad Amin Shayestehpour, Øystein Bjelland, Martin G. Gregersen, Håvard Furunes and Marius Molund in Foot & Ankle Orthopaedics

## Appendix

**Table S1.** Defined Joints in the Model and Their Properties.

Joint Name	Joint Type	Reaction Force Enabled^a^	FDK DOF	Joint Segments
Talocrural joint	Revolute joint	Ty, Rz	Tx, Tz, Rx, Ry	Talus, tibia
Subtalar joint	Revolute joint	Ty	Tx, Tz, Rx, Ry, Rz	Foot, talus
Tibiofibular joint	Fixed joint	–	Tx, Ty, Tz, Rx, Ry, Rz	Tibia, fibula
Calcaneus-ground joint	Fixed (rigid) joint	Tx, Ty, Tz, Rx, Ry, Rz	–	Foot, ground

Abbreviation: FDK DOF, degrees of freedom enabled for force-dependent kinematics.

a Reaction force–enabled directions indicate which DOFs are active in constraint reactions. Tx, Ty, and Tz denote translations; Rx, Ry, and Rz denote rotations.

**Table S2.** Tibiofibular Joint Stiffness Parameters.

Parameter	Stiffness	Unit
Rotational stiffness about local X axis	2500	N/radian
Rotational stiffness about local Y axis	500	N/radian
Rotational stiffness about local Z axis	1000	N/radian
Linear stiffness in local X axis	7000	N/mm
Linear stiffness in local Y axis	5000	N/mm
Linear stiffness in local Z axis	10 000	N/mm

**Table S3.** Ligament Properties.

Ligament	Initial Length, mm	Stiffness, N/mm	Cross-Sectional Area, mm²
ATFL	13.8	25.2	36
PTFL	17.1	13.6	33
CFL	27.9	4.1	16
DPTTL	12.2	33.0	57
TCL	20.0	12.7	49
SPTTL	30.3	11.4	36^a^
TNL	34.5	7.4	36^a^
DATTL	11.6	22.0	36^a^
TSL	45.7	7.0	36^a^
PTCL	16.2	10.0	36^a^

Initial lengths are measured from the neutral state in an unloaded model.

a In absence of reported cross-sectional area data, some ligaments were assigned to the average cross-sectional area of the ATFL.

**Table S4.** Identified Stiffness Parameters for Talocrural and Subtalar Joints.

Parameter	Stiffness	Unit
Talocrural rotational stiffness about local Y axis	38.26	N/radian
Talocrural rotational stiffness about local X axis	0.00	N/radian
Talocrural translational stiffness in local X axis	5403.54	N/mm
Talocrural translational stiffness in local Z axis	10 000.0	N/mm
Subtalar rotational stiffness about local Z axis	2623.91	N/radian
Subtalar rotational stiffness about local Y axis	301.73	N/radian
Subtalar rotational stiffness about local X axis	3986.59	N/radian
Subtalar translational stiffness in local X axis	1943.75	N/mm
Subtalar translational stiffness in local Z axis	10 000.0	N/mm
